# New-Onset Chronic Musculoskeletal Pain Following COVID-19 Infection Fulfils the Fibromyalgia Clinical Syndrome Criteria: A Preliminary Study

**DOI:** 10.3390/biomedicines12091940

**Published:** 2024-08-23

**Authors:** Omar Khoja, Matthew Mulvey, Sarah Astill, Ai Lyn Tan, Manoj Sivan

**Affiliations:** 1 Leeds Institute of Rheumatic and Musculoskeletal Medicine, University of Leeds, Leeds LS7 4SA, UK; umok@leeds.ac.uk (O.K.); a.l.tan@leeds.ac.uk (A.L.T.); 2 Academic Unit of Palliative Care, Leeds Institute of Health Sciences, University of Leeds, Leeds LS2 9NL, UK; m.r.mulvey@leeds.ac.uk; 3 School of Biomedical Sciences, Faculty of Biological Sciences, University of Leeds, Leeds LS2 9JT, UK; s.l.astill@leeds.ac.uk; 4 NIHR Leeds Biomedical Research Centre, Leeds Teaching Hospitals NHS Trust, Leeds LS7 4SA, UK; 5 COVID Rehabilitation Service, Leeds Community Healthcare NHS Trust, Leeds LS11 0DL, UK

**Keywords:** long COVID, post-COVID-19 condition, post-acute sequela, FM syndrome, chronic pain, chronic widespread pain, post-acute infection syndrome (PAIS)

## Abstract

New-onset chronic musculoskeletal (MSK) pain (>3 months duration) is a common symptom of post-COVID-19 syndrome (PCS). This study aimed to characterise new-onset chronic MSK pain in patients with PCS and its overlap with Fibromyalgia Syndrome (FMS). We enrolled patients with new-onset chronic MSK pain post-COVID-19 and assessed the nature of the pain and associated symptoms using the C19-YRS (Yorkshire Rehabilitation Scale). The FMS assessment was conducted as part of a standard clinical examination using the American College of Rheumatology (ACR) 2010 criteria: (1) Widespread Pain Index (WPI) ≥ 7 and symptoms severity (SS) score ≥ 5, or WPI between 3 and 6 and SS score ≥ 9, (2) symptoms consistent for at least 3 months, and (3) no alternative diagnosis. Of the eighteen patients (average age 49.6 (SD 11.8) years; BMI 31.7 (SD 8.6)), twelve were female. The average symptom duration was 27.9 (SD 6.97) months post-infection. Thirteen patients (72.2%) met the FMS criteria, with an average WPI score of 8.8 and an average SS score of 8.2, indicating a high level of pain and significant quality of life impacts. These findings support the hypothesis that FMS may develop as a long-term sequela of a viral infection, underscoring the need for further research into post-viral long-term conditions.

## 1. Introduction

Post-COVID-19 syndrome (PCS) involves symptoms that persist beyond twelve weeks following an acute SARS-CoV-2 infection [1]. PCS is a novel, multifaceted condition that presents a significant challenge to healthcare systems globally. It is estimated that a minimum of 10% of individuals who recover from COVID-19 develop PCS, with the prevalence increasing to 30% among moderate non-hospitalised cases and up to 70% in those who are hospitalised [2]. In the United Kingdom alone, approximately two million people are currently experiencing symptoms of PCS, highlighting its extensive impact [3]. One of the common and enduring symptoms of PCS is new-onset chronic Musculoskeletal (MSK) pain [4,5]. This adds a considerable strain to the already substantial global burden of chronic MSK pain, estimated to affect 1.7 billion individuals worldwide before the COVID-19 pandemic [6]. New-onset MSK pain in PCS is associated with a wide array of symptoms, including fatigue, sleep disturbances, and cognitive issues. These symptoms are similar to those observed in Fibromyalgia Syndrome (FMS) [7,8,9].

Fibromyalgia Syndrome is a complex and chronic condition characterised by widespread pain, fatigue, sleep problems, and cognitive disturbance, which significantly impair quality of life [10]. It affects between 2% and 8% of the general population, with a higher prevalence in females and middle-aged individuals [11]. FMS is widely recognised as being associated with central and peripheral nervous system sensitisation [12,13]. This results in pain that often manifests as widespread aching and burning. Fibromyalgia Syndrome has been observed alongside other chronic pain conditions such as osteoarthritis and rheumatoid arthritis. Notably, about 10% to 30% of patients with rheumatic disorders also meet the criteria for FMS, often referred to as secondary fibromyalgia [14]. Despite the high prevalence of FMS and its well-documented clinical characteristics, its precise aetiology remains largely unclear. A few studies have shed light on potential triggers for the development of FMS, pointing toward inflammatory and autoimmune disorders, as well as viral infections, as possible contributing factors [15,16]. The association between FMS and specific infections has been studied, revealing links with various infection agents, including Epstein—Barr virus, Q fever, viral hepatitis, HIV, and Lyme disease [16,17].

Recent research has identified a notable overlap in the symptoms and pathophysiological mechanisms of PCS and FMS, suggesting that the SARS-CoV-2 virus could play a pivotal role in mediating FMS and expanding our understanding of how infections might trigger its development [8,9,18,19]. This emerging evidence points to a substantial occurrence of FMS among those recovering from COVID-19, underlining the need for further investigation into the relationship between infectious diseases and chronic pain syndromes.

The aim of this study was to characterise the nature of new-onset chronic MSK pain in individuals with PCS and explore its commonalities with FMS, utilising validated diagnostic tools and outcome measures. This research aimed to identify similarities in characteristics between PCS and FMS that could reveal underlying mechanisms and inform more effective treatment strategies for chronic MSK pain in individuals with PCS.

## 2. Materials and Methods

### 2.1. Study Design and Participants

In this observational study, we assessed the characteristics of new-onset MSK pain and investigated its overlap with FMS. These data were derived from the Musculoskeletal Pain in Long COVID (MUSLOC) study, a prospective longitudinal study (clinicaltrials.gov NCT05358119). Participants eligible for this study met the following criteria: they were 18 years or older, had tested positive for COVID-19 or had COVID-19 symptoms confirmed by an independent clinician, received a clinical diagnosis of PCS according to the NICE guidelines, experienced new-onset MSK pain since their COVID-19 infection, were willing to adhere to study procedures, and could understand and read English. We excluded patients with pre-existing chronic MSK pain prior to their COVID-19 infection.

The Leeds Long COVID Community Rehabilitation Service acted as the primary recruitment site, supplemented by advertisements on social media, support groups, and research websites for wider outreach. Potential participants expressing interest were first contacted via telephone for eligibility screening. During this initial contact, they were fully briefed on the study’s requirements, including potential disadvantages and benefits, before providing their written informed consent. This research adheres to the ethical principles of the Declaration of Helsinki and was approved by the London–Central Research Ethics Committee (REC), the Health Research Authority (HRA), and Health and Care Research Wales (HCRW) (Ref 21/PR/1377).

### 2.2. Procedure and Outcomes

Demographic information including age, sex, ethnicity, marital status, body mass index (BMI), pre-existing comorbidities, date of COVID-19 infection, hospitalisation due to COVID-19, number and dates of COVID-19 vaccinations, employment category, and whether their employment status had been affected by the COVID-19 pandemic and PCS was collected.

PCS-associated symptoms were assessed using the COVID-19 Yorkshire Rehabilitation Scale (C19-YRS) questionnaire [20]. It comprises a 0–10 Numerical Rating Scale (NRS) of the main symptoms of PCS (including breathlessness, cough, swallowing, fatigue, continence, pain, cognition, post-traumatic stress disorder (PTSD), anxiety, depression, palpitations, dizziness, weakness, sleep problems, fever, and skin rash) and their impact on five daily functions (including communication, mobility, personal care, activities of daily living, and social role). Participants were also asked to grade their symptoms prior to contracting COVID-19 infection (pre-COVID-19). The score of items 1–10 adds up to the Symptoms Severity score (0–100), while the score of items 11–15 adds up to the functional disability score (0–50). The C19-YRS also includes a question regarding overall health status before and after COVID-19 infection (scores 0–10), in which a score of 0 means the WORST health the respondent can imagine and a score of 10 means the BEST health they can imagine. 

The assessment of FMS was conducted as part of the standard clinical examination in MUSLOC using the American College of Rheumatology (ACR) 2010 criteria [21]. It evaluates the Widespread Pain Index (WPI) by identifying 19 body areas where patients have experienced pain in the previous week. Additionally, the ACR criteria examine symptoms severity (SS) across two parts: Part 1 quantifies the severity of fatigue, waking unrefreshed, and cognitive symptoms on a scale from 0 (no problem) to 3 (severe problems). Part 2 evaluates a broader spectrum of 40 other somatic symptoms, categorised as follows: no symptoms (score of 0), 1 to 10 symptoms (score of 1), 11 to 24 symptoms (score of 2), and 25 or more symptoms (score of 3) [22]. The criteria for diagnosing FMS are satisfied if all three of the following conditions are met: (1) WPI score ≥ 7 and SS score ≥ 5, or WPI score 3–6 and SS score ≥ 9, (2) symptoms have been present at a similar level for at least 3 months, and (3) the patient does not have a disorder that would otherwise explain the pain.

### 2.3. Statistical Analysis

Statistical analysis was performed using the IBM Statistical Package for the Social Sciences (SPSS) software version 29. Descriptive statistics are expressed as the mean, standard deviation, minimum and maximum as appropriate for continuous variables, while numbers and frequencies are used for categorical variables. A paired sample *t*-test was utilised to assess the variation in C19-YRS symptoms severity, functional disability, and global health scores.

## 3. Results

### 3.1. Patient Characteristics

A total of 18 patients were included in the study. The mean age of the patients was 49.6 (SD 11.8) years, comprising 12 females (66.7%) and 6 (33.3%) males. The body mass index (BMI) was 31.7 (SD 8.6). The mean duration of the onset of COVID-19 infection to the data collection point was 27.9 (SD 6.97) months. 

### 3.2. Pain Characteristics

Seventeen (94.4%) patients reported experiencing myalgia; of these, fifteen (83.3%) also experienced arthralgia. Fourteen (77.8%) patients reported experiencing generalised widespread pain. The remaining patients, who did not report widespread pain, still experienced pain in at least four distinct body areas. Table 1 summarises the demographic characteristics and distribution of general pain locations.

### 3.3. Results of C19-YRS

The data from C19-YRS showed the new-onset (de novo) of PCS symptoms compared to pre-COVID. All participants experienced pain and fatigue, while breathlessness was noted by 17 (94.4%) of them, predominantly during physically demanding activities such as stair climbing. Cognitive issues and anxiety were also prevalent symptoms, reported by 93% and 89% of the cohort, respectively. Additionally, fifteen patients (83.3%) reported having sleep problems.

There was also a significant increase in functional disability scores compared to levels before infection. PCS symptoms interfered with daily living activities for 17 (94.4%) patients, impacting their ability to perform family care and maintain social interactions. Many patients also reported detrimental effects on their communication abilities (15 patients), mobility (12 patients), and personal care (11 patients), underscoring a substantial burden on their daily lives compared to their pre-COVID-19 status. Patients also observed a notable deterioration in their general health after contracting COVID-19, with overall health ratings decreasing from a pre-COVID-19 average of 8.1 (SD 1.1) to 3.0 (SD 1.8) post-infection. The changes in symptom severity before and after COVID-19 infection are detailed in Table 2.

### 3.4. Results of ACR 2010 Criteria for FMS

Thirteen (72.2%) of the evaluated patients met the diagnostic criteria for FMS as defined by the ACR. The average WPI score among the patients was 8.8 (SD 5.1), indicating a high level of pain spread across multiple body regions. Additionally, the average SS score was 8.2 (SD 2.4), reflecting significant symptom severity related to fatigue, waking unrefreshed, cognitive symptoms, and the extent of other somatic symptoms. These scores collectively suggest a substantial impact on patients’ quality of life and align with the typical symptomatology observed in FMS.

The remaining five patients of the study group, representing 27.8%, did not meet the ACR criteria for FMS, with four of these five patients being males. The reasons for not meeting the ACR criteria varied and were related to their WPI and SS scores. Specifically, one participant had a WPI of 8 but an insufficient SS of 4, failing to satisfy criterion 1a, indicating that while they experienced widespread pain, the severity of their symptoms was not adequately high. Two participants, one with a WPI of 5 and SS of 7 and another with a WPI of 5 and SS of 6, met the WPI required for criterion 1b, but their SS scores were too low; thus, they did not meet the FMS criteria. Additionally, two participants with scores of WPI 0 and SS 3 and WPI 1 and SS 9, respectively, also failed to meet the criteria. The latter had a high SS score, indicating severe symptoms, yet the extremely low WPI made them ineligible for the FMS diagnostic criteria.

## 4. Discussion

In this study, we explored the characteristics of new-onset MSK pain in individuals with PCS and its similarities with FMS. The research aimed to identify overlaps between PCS and FMS that could reveal underlying mechanisms and inform more effective strategies for both chronic MSK pain in individuals with PCS and FMS. Our findings revealed that a substantial number of participants (72.2%) met the ACR criteria for FMS, underscoring a significant overlap between PCS and FMS. This demonstrates a high prevalence of widespread pain and symptomatology consistent with FMS in the PCS population.

The findings of this study align with and expand upon recent research exploring the connection between FMS and the SARS-CoV-2 virus, enhancing our understanding of how viral infections may trigger chronic long-term conditions such as FMS. For instance, Haider et al. studied 203 PCS patients, identifying a subset whose symptoms closely resemble FMS, negatively impacting cognitive and physical function. Their data also revealed that 8.4% of those patients met the ACR criteria for FMS. Notably, the study also included another group of 22 patients who had already been diagnosed with both PCS and FMS, underscoring a significant overlap in the symptomatology of both conditions [9]. Additionally, Ursini et al. reported that 30.7% of individuals with PCS meet the FMS diagnostic criteria, suggesting a substantial prevalence of FMS manifestation among those who recovered from COVID-19 [18]. In another study, Savin et al. investigated the incidence of FMS in 198 patients who were discharged after COVID-19 hospitalisation, which was found to be 15%, with a higher prevalence in females (63%) [23]. These are considerably lower than the 72.2% identified in our study, indicating varying levels of FMS manifestations across different cohorts and the different criteria used for diagnosis. However, our results align with the existing evidence that FMS is more prevalent among females. Additionally, Fialho et al. highlighted the similarities in the pathophysiological mechanisms and symptoms of FMS and PCS, suggesting a potential mediating role of the SARS-CoV-2 virus in the development of FMS [8]. These studies collectively highlight the complex relationship between PCS and FMS and suggest a potential viral contribution to the chronicity of MSK pain, aligning with our findings of significant symptom overlap and functional impairment in PCS, similar to FMS patterns.

There is a growing body of evidence suggesting the link between infections and FMS (Table 3). Studies have investigated the long-term sequelae after several viral and other infections and applied FMS diagnostic criteria to follow-up assessments (similar to our study). Different criteria were used, including the 2010 ACR criteria and the 2016 modified criteria. Some studies that included control groups clearly demonstrate the emergence of FMS after infection, which supports the main hypothesis of our study. The 2016 modified criteria increase the likelihood of a diagnosis as it emphasises that FMS is not a diagnosis of exclusion and needs to be diagnosed if the criteria are met (irrespective of other contributing conditions/factors) [24].

Although the connection between the SARS-CoV-2 virus and FMS is still under investigation, several hypothetical mechanistic connections may be suggested based on what we know about the pathogenesis of these two different conditions. There might be similar pathophysiological pathways involved, as the virus affects both the central and peripheral nervous systems, potentially leading to typical FMS symptoms like pain and sensory disturbances either by direct tissue damage or systemic inflammatory responses [8,9,23]. Additionally, the chronic stress and psychological impacts of the pandemic, such as isolation and ongoing anxiety, can exacerbate the central sensitisation seen in both conditions [31,32]. Finally, the intense inflammatory response, often referred to as a “cytokine storm”, which is triggered by the SARS-CoV-2 virus, can lead to chronic inflammation that affects the nervous system. This sustained inflammatory state could potentially initiate or aggravate FMS symptoms, underscoring a plausible link between COVID-19 and new-onset or worsening FMS [15,16].

This study has several limitations, including the absence of a control group and reliance on patient-reported questionnaires. We, however, need to acknowledge that these conditions are clinical syndromes, and their assessment is entirely reliant on subjective symptoms. The other limitation could be that those who did not meet the FMS criteria were assessed on a “good” day, given that PCS is a fluctuating condition with good days (with less severity of the condition) and bad days (worse severity of the condition) [33]. The relatively small patient group may also limit the generalisability of our findings. Despite these constraints, this study utilised validated diagnostic tools to rigorously assess the diagnostic criteria overlap, highlight the potential of shared mechanisms, and provide critical insights into our understanding of how chronic widespread pain syndromes could evolve after an infection. 

Future research should involve a larger sample size, a control group (including those who had COVID-19 infection but no PCS symptoms) and follow both groups longitudinally with multiple assessment points (to incorporate the fluctuations seen in the conditions). We also need large mechanistic studies to better understand the long-term impacts of persistent PCS and the development of FMS in individuals with PCS. Additionally, the growing body of evidence underscores the need for a deeper investigation into the pathophysiological mechanisms of long-term syndromes after any infection, which could revolutionise approaches to diagnosis and the treatment of post-infection sequelae.

## 5. Conclusions

Our study shows that new-onset chronic MSK pain following COVID-19 infection often meets the diagnostic criteria for FMS. These findings support the hypothesis that FMS can arise as a sequela of viral infections. Given these findings, it is critical to consider and screen for FMS in patients presenting with persistent PCS symptoms, particularly chronic pain and fatigue. This understanding not only challenges the current vagueness in validating PCS and FMS but also emphasises the need for further investment in clinical services and research to investigate the underlying mechanisms through which viral infections could lead to chronic conditions like FMS. The appropriate validation and management of FMS could potentially enhance patients’ understanding of the condition and the outcomes seen in services managing these conditions.

## Figures and Tables

**Table 1:** Demographic and pain characteristics of patients with new-onset chronic MSK pain after COVID-19 infection in Leeds, UK, 2023–2024.

Full Sample (n = 18)	n	%
Age, years		
<30	1	5.6
30–39	3	16.7
40–49	4	22.2
50–59	6	33.3
60+	4	22.2
Sex		
Female	12	66.7
Male	6	33.3
BMI (kg/m^2^)	Mean 31.7 (±8.6)	
Ethnicity		
White	14	77.8
Asian	4	22.2
Black/mixed/other	0	00.0
Duration of LC symptoms (months)		
<12	0	0.00
13–24	7	38.9
25–36	8	44.4
37–48	3	16.7
+49	0	0
Number of COVID-19 infections		
Infected once	4	22.2
Infected twice	10	55.6
Infected 3 times	3	16.7
Infected 4 times	1	5.6
Marital status		
Single	4	22.2
Married or in a domestic relationship	14	77.8
Smoking status		
None	17	94.4
Active	1	5.6
Comorbidities reported by patients (pre-existing before COVID-19 infection) *		
Diabetes (type 1 or 2)	1	5.6
Hypertension	2	11.1
Chronic respiratory conditions	3	16.7
Chronic heart disease	2	11.1
Chronic neurological conditions	2	11.1
History of chronic musculoskeletal conditions	2	11.1
Chronic kidney disease	0	0
Chronic liver disease	0	0
Do not have any previous medical conditions	6	33.3
Pain Frequency		
Intermittent (episodic)	4	22.2
Continuous	14	77.8
Quality of pain *		
Dull	14	77.8
Acing	15	83.3
Sharp and stabbing	5	27.8
Throbbing	4	22.2
Burning	4	22.2
Numbness	3	16.7
Crushing	2	11.1
Stinging	0	0
Pain location reported by participants *		
Widespread pain	14	77.8
Upper extremity	11	61.1
Lower extremity	14	77.8
Spine	12	66.7
MSK chest pain	4	22.2
* Total percentages may exceed 100% as patients could report multiple responses.		

**Table 2:** Summary of C19-YRS findings for patients with new-onset chronic MSK pain after COVID-19 infection in Leeds, UK, 2023–2024.

C19-YRS Items	Pre-COVID-19	Post-COVID-19
Main symptoms of LC		
1. Breathlessness in number of participants (% of participants)	8 (44.4%)	17 (94.4%)
At rest (mean severity, SD)	0.84 (1.54)	2.50 (2.87)
Dressing (mean severity, SD)	0.88 (2.34)	3.94 (3.17)
Stairs (mean severity, SD)	0.76 (1.71)	5.89 (3.22)
2. Cough/throat sensitivity/voice change (% of participants)	3 (16.7%)	9 (50.0%)
(mean severity, SD)	0.76 (1.99)	2.72 (3.08)
3. Swallowing/nutrition (% of participants)	1 (5.6%)	4 (22.2%)
(mean severity, SD)	0.24 (0.97)	0.98 (1.81)
4. Fatigue (% of participants)	12 (66.7%)	18 (100%)
(mean severity, SD)	1.47 (1.18)	7.50 (1.98)
5. Continence (% of participants)	4 (22.2%)	6 (33.3%)
(mean severity, SD)	0.53 (1.28)	2.33 (3.46)
6. Chronic pain/discomfort (% of participants)	0 (0.0%)	18 (100%)
(mean severity, SD)	0.00 (0.00)	6.61 (2.30)
7. Cognition (% of participants)	8 (44.4%)	17 (94.4%)
(mean severity, SD)	1.35 (1.77)	6.56 (2.71)
8. Anxiety (% of participants)	12 (66.7%)	16 (88.9%)
(mean severity, SD)	2.00 (1.84)	5.00 (3.24)
9. Depression (% of participants)	6 (33.3%)	14 (77.8%)
(mean severity, SD)	1.24 (2.08)	4.22 (2.92)
10. PTSD (% of participants)	3 (16.7%)	9 (50.0%)
(mean severity, SD)	0.41 (1.23)	2.44 (2.79)
Symptoms severity score (0–100)	8.88 (9.37)	40.78 (17.26)
* *p* value	<0.001	
11. Communication (% of participants)	5 (27.8%)	15 (83.3%)
(mean, SD)	0.53 (0.94)	4.06 (2.60)
12. Mobility (% of participants)	5 (27.8%)	12 (66.7%)
(mean, SD)	0.53 (1.23)	4.06 (3.67)
13. Personal Care (% of participants)	2 (11.1%)	11 (61.1%)
(mean, SD)	0.41 (1.46)	2.78 (3.28)
14. Other Activities of Daily Living (% of participants)	4 (22.2%)	17 (94.4%)
(mean, SD)	0.41 (1.46)	6.6 (2.6)
15. Social role (% of participants)	2 (11.1%)	15 (83.3%)
(mean, SD)	0.12 (0.33)	5.17 (2.78)
Functional disability score (0–50)	2.00 (3.54)	22.33 (12.55)
* *p* value	<0.001	
Global health (mean, SD) (0–10) ‘10 Best Health’	8.13 (1.09)	3.00 (1.79)
* *p* value	<0.001	
* Paired sample *t*-test evaluated changes in symptom severity, fuctional disability, and global health scores.		

**Table 3:** Incidence of fibromyalgia following different acute infections.

Infection	Type	Study	Findings (Diagnosis of FM)
SARS CoV2 (COVID-19)	RNA virus	616 individuals with COVID-19 infection history completing a questionnaire with 2010 ACR criteria [18]	30.7% met the criteria for FMS at 6 m
		404 individuals with COVID-19 infection history completing a questionnaire with 2010 ACR criteria [25]	19.8% met the criteria for FMS (average FU duration not mentioned)
		404 individuals with a COVID-19 infection history completed a questionnaire with 2016 ACR criteria [26]	40% met the criteria for FMS (average FU duration not mentioned)
		198 individuals with COVID-19 infection history completing a questionnaire with 2016 ACR criteria [23]	15% met the criteria for FMS at 5 m
Hepatitis C	RNA virus	90 patients with HCV and 128 anti-HCV negative controls; used 1990 criteria [16]	16% of HCV group; 0% healthy controls; 92% were FMS-positive in HCV group female
Hepatitis B	DNA virus	50 HBsAg-positive Ab-negative and 50 HBsAg-negative healthy volunteers; matched for age/sex/BMI/AST/ALT; used 1990 criteria [27]	26% of HBsAg; 4% of controls (*p* < 0.001); 92% FMS-positive in HBsAg group female
HTLV-I	Retrovirus	100 HTLV-1-positive and 62 HTLV-1-negative blood donors [28]	38% HTLV-1 group; 4.8% controls; *p* = 0.008
Giardia Lamblia	Protozoon	572 Giardia-positive case and 673 controls; used 2016 FMS criteria [29]	OR = 2.91; *p* < 0.001
Lyme (Borrelia Burgdoferi)	Bacterium	Meta-analysis of 5 studies involving 504 patients and 530 controls, capturing symptoms [30]	FM-type symptoms; *p* < 0.00001

## Data Availability

The data presented in this study are available on request from the corresponding author. The data are not publicly available due to privacy concerns.

## References

[1] National Institute for Health and Care Excellence (NICE), Scottish Intercollegiate Guidelines Network (SIGN), Royal College of General Practitioners (RCGP) (2022). COVID-19 Rapid Guideline: Managing the Long-term Effects of COVID-19. https://www.nice.org.uk/guidance/ng188.

[2] Bull-Otterson L., Baca S., Saydah S., Boehmer T.K., Adjei S., Gray S., Harris A.M. (2022). Post-COVID Conditions Among Adult COVID-19 Survivors Aged 18–64 and ≥65 Years. MMWR Morb. Mortal. Wkly. Rep..

[3] Ayoubkhani D., Bosworth M., King S. Prevalence of ongoing symptoms following coronavirus (COVID-19) infection in the UK: 30 March 2023. https://www.ons.gov.uk/peoplepopulationandcommunity/healthandsocialcare/conditionsanddiseases/bulletins/prevalenceofongoingsymptomsfollowingcoronaviruscovid19infectionintheuk/30march2023.

[4] Khoja O., Passadouro B.S., Mulvey M., Delis I., Astill S., Tan A.L., Sivan M. (2022). Clinical Characteristics and Mechanisms of Musculoskeletal Pain in Long COVID. J. Pain Res..

[5] Fernández-De-Las-Peñas C., Navarro-Santana M.P., Plaza-Manzano G., Palacios-Ceña D., Arendt-Nielsen L.D.M. (2021). Time course prevalence of post-COVID pain symptoms of musculoskeletal origin in patients who had survived severe acute respiratory syndrome coronavirus 2 infection: A systematic review and meta-analysis. Pain.

[6] Cieza A., Causey K., Kamenov K., Hanson S.W., Chatterji S., Vos T. (2021). Global Estimates of the Need for Rehabilitation Based on the Global Burden of Disease Study 2019: A Systematic Analysis for the Global Burden of Disease Study 2019. Lancet.

[7] Khoja O., Silva-Passadouro B., Cristescu E., McEwan K., Doherty D., O’Connell F., Ponchel F., Mulvey M., Astill S., Tan A.L. (2024). Clinical Characterization of New-Onset Chronic Musculoskeletal Pain in Long COVID: A Cross-Sectional Study. J. Pain Res..

[8] Fialho M.F.P., Brum E.S., Oliveira S.M. (2023). Could the Fibromyalgia Syndrome Be Triggered or Enhanced by COVID-19?. Inflammopharmacology.

[9] Haider S., Janowski A.J., Lesnak J.B., Hayashi K., Dailey D.L., Chimenti R., Frey-Law L.A., Sluka K.A., Berardi G. (2023). A Comparison of Pain, Fatigue, and Function between Post-COVID-19 Condition, Fibromyalgia, and Chronic Fatigue Syndrome: A Survey Study. Pain.

[10] Clauw D.J. (2014). Fibromyalgia: A Clinical Review. JAMA.

[11] Borchers A.T., Gershwin M.E. (2015). Fibromyalgia: A Critical and Comprehensive Review. Clin. Rev. Allergy Immunol..

[12] Woolf C.J. (2011). Central sensitization: Implications for the diagnosis and treatment of pain. Pain.

[13] Williams D.A., Clauw D.J. (2009). Understanding Fibromyalgia: Lessons from the Broader Pain Research Community. J. Pain.

[14] Phillips K., Clauw D.J. (2013). Central Pain Mechanisms in the Rheumatic Diseases: Future Directions. Arthritis Rheum..

[15] Siracusa R., Di Paola R., Cuzzocrea S., Impellizzeri D. (2021). Fibromyalgia: Pathogenesis, Mechanisms, Diagnosis and Treatment Options Update. Int. J. Mol. Sci..

[16] Buskila D., Atzeni F., Sarzi-Puttini P. (2008). Etiology of Fibromyalgia: The Possible Role of Infection and Vaccination. Autoimmun. Rev..

[17] Ablin J.N., Shoenfeld Y., Buskila D. (2006). Fibromyalgia, Infection and Vaccination: Two More Parts in the Etiological Puzzle. J. Autoimmun..

[18] Ursini F., Ciaffi J., Mancarella L., Lisi L., Brusi V., Cavallari C., D’onghia M., Mari A., Borlandelli E., Cordella J.F. (2021). Fibromyalgia: A new facet of the post-COVID-19 syndrome spectrum? Results from a web-based survey. RMD Open.

[19] Silva-Passadouro B., Tamasauskas A., Khoja O., Casson A.J., Delis I., Brown C., Sivan M. (2024). A Systematic Review of Quantitative EEG Findings in Fibromyalgia, Chronic Fatigue Syndrome and Long COVID. Clin. Neurophysiol..

[20] Sivan M., Halpin S., Gee J. (2020). Assessing Long-Term Rehabilitation Needs in COVID-19 Survivors Using a Telephone Screening Tool (C19-YRS Tool). Adv. Clin. Neurosci. Rehabil..

[21] Wolfe F., Clauw D.J., Fitzcharles M.-A., Goldenberg D.L., Katz R.S., Mease P., Russell A.S., Russell I.J., Winfield J.B., Yunus M.B. (2010). The American College of Rheumatology Preliminary Diagnostic Criteria for Fibromyalgia and Measurement of Symptom Severity. Arthritis Care Res..

[22] Galvez-Sánchez C.M., Reyes Del Paso G.A. (2020). Diagnostic Criteria for Fibromyalgia: Critical Review and Future Perspectives. J. Clin. Med..

[23] Savin E., Rosenn G., Tsur A.M., Hen O., Ehrenberg S., Gendelman O., Buskila D., Halpert G., Amital D., Amital H. (2023). The Possible Onset of Fibromyalgia Following Acute COVID-19 Infection. PLoS ONE.

[24] Wolfe F., Clauw D.J., Fitzcharles M.A., Goldenberg D.L., Häuser W., Katz R.L., Mease P.J., Russell A.S., Russell I.J., Walitt B. (2016). 2016 Revisions to the 2010/2011 Fibromyalgia Diagnostic Criteria. Semin. Arthritis Rheum..

[25] Akel A., Almanasyeh B., Kobaa A.A., Aljabali A., Al-Abadleh A., Alkhalaileh A., Alwardat A.R., Sarhan M.Y., Abu-Jeyyab M. (2023). A Cross-Sectional Study of Fibromyalgia and Post-acute COVID-19 Syndrome (PACS): Could There Be a Relationship?. Cureus.

[26] Bileviciute-Ljungar I., Norrefalk J.-R., Borg K. (2022). Pain Burden in Post-COVID-19 Syndrome Following Mild COVID-19 Infection. J. Clin. Med..

[27] Adak B., Tekeoǧlu Í., Ediz L., Budancamanak M., Yazgan T., Karahocagil K., Demirel A. (2005). Fibromyalgia Frequency in Hepatitis B Carriers. J. Clin. Rheumatol..

[28] Cruz B.A., Catalan-Soares B., Proietti F. (2006). Higher Prevalence of Fibromyalgia in Patients Infected with Human T Cell Lymphotropic Virus Type I. J. Rheumatol..

[29] Hunskar G.S., Rortveit G., Litleskare S., Eide G.E., Hanevik K., Langeland N., Wensaas K.A. (2022). Prevalence of Fibromyalgia 10 Years after Infection with Giardia Lamblia: A Controlled Prospective Cohort Study. Scand. J. Pain.

[30] Cairns V., Godwin J. (2005). Post-Lyme Borreliosis Syndrome: A Meta-Analysis of Reported Symptoms. Int. J. Epidemiol..

[31] Colas C., Jumel A., Vericel M.-P., Barth N., Manzanares J., Goutte J., Fontana L., Féasson L., Hupin D., Guyot J. (2021). Understanding Experiences of Fibromyalgia Patients Involved in the Fimouv Study During COVID-19 Lockdown. Front. Psychol..

[32] Qi T., Hu T., Ge Q.Q., Zhou X.N., Li J.M., Jiang C.L., Wang W. (2021). COVID-19 Pandemic Related Long-Term Chronic Stress on the Prevalence of Depression and Anxiety in the General Population. BMC Psychiatry.

[33] Sivan M., Smith A.B., Osborne T., Goodwin M., Lawrence R.R., Baley S., Williams P., Lee C., Davies H., Balasundaram K. (2024). Long COVID Clinical Severity Types Based on Symptoms and Functional Disability: A Longitudinal Evaluation. J. Clin. Med..

